# 4225 Evaluation of Neurotransmitters in Channelopathy-Related Epilepsy

**DOI:** 10.1017/cts.2020.298

**Published:** 2020-07-29

**Authors:** Sunita N Misra, Theresa M. Czech, Jennifer A. Kearney

**Affiliations:** 1 Northwestern University

## Abstract

OBJECTIVES/GOALS: Variants in voltage-gated sodium channels (VGSC) are a common cause of severe early onset epilepsy. Changes in CSF neurotransmitters (NT) were identified in 2 cases of VGSC-related epilepsy. Here we investigate NT changes in patients and a novel mouse model of VGSC-related epilepsy. METHODS/STUDY POPULATION: We conducted a single site IRB approved retrospective chart review of patients with VGSC-related epilepsy who underwent CSF NT testing for diagnostic purposes. In parallel, we examined NT levels from the brains of wild-type (WT) and a novel VGSC-related epilepsy mouse model after obtaining IACUC approval. We rapidly isolated forebrain, cortex, striatum, and brainstem from 5-6 animals per sex and genotype. A combination of HPLC with electrochemical detection and mass spectrometry were used to quantify NT levels from brain samples. RESULTS/ANTICIPATED RESULTS: We identified 10 patients with VGSC-related epilepsy who received CSF NT testing. Two of these patients had abnormal NT results including changes to dopamine (DA) or serotonin (5-HT) metabolites. We analyzed NT levels from four brain regions from male and female WT and VGSC-related epilepsy mice. We anticipate that most of the NT levels will be similar to WT, however subtle changes in the DA or 5-HT metabolites may be seen in VGSC-related epilepsy. DISCUSSION/SIGNIFICANCE OF IMPACT: Patients with VGSC-related epilepsy often have autism spectrum disorder, sleep, and movement disorders. Understanding the role of aberrant NT levels in VGSC-related epilepsy may provide additional therapeutic targets that address common neuropsychological comorbidities as well as seizures.

